# Recoil Measurements in *Drosophila* Embryos: from Mounting to Image Analysis

**DOI:** 10.21769/BioProtoc.4806

**Published:** 2023-07-20

**Authors:** Luis Eduardo Sánchez-Cisneros, Sourabh Bhide, Luis Daniel Ríos-Barrera

**Affiliations:** 1 Departamento de Biología Celular y Fisiología, Instituto de Investigaciones Biomédicas, Universidad Nacional Autónoma de México, Mexico City, Mexico; 2 European Molecular Biology Laboratory, Heidelberg, Germany; 3 GSK, Heidelberg, Germany

**Keywords:** Laser cuts, Recoil measurements, Tension measurements, Laser microdissection, Biomechanics, Particle image velocimetry

## Abstract

Tension and force propagation play a central role in tissue morphogenesis, as they enable sub- and supra-cellular shape changes required for the generation of new structures. Force is often generated by the cytoskeleton, which forms complex meshworks that reach cell–cell or cell–extracellular matrix junctions to induce cellular rearrangements. These mechanical properties can be measured through laser microdissection, which concentrates energy in the tissue of interest, disrupting its cytoskeleton. If the tissue is undergoing tension, this cut will induce a recoil in the surrounding regions of the cut. This protocol describes how one can perform laser microdissection experiments and subsequently measure the recoil speed of the sample of interest. While we explain how to carry out these experiments in *Drosophila embryos*, the recoil calibration and downstream analyses can be applied to other types of preparations.

Key features

Allows measuring tension in live *Drosophila* embryos with a relatively simple approach.

Describes a quick way to mount a high number of embryos.

Includes a segmentation-free recoil quantification that reduces bias and speeds up analysis.

Graphical overview

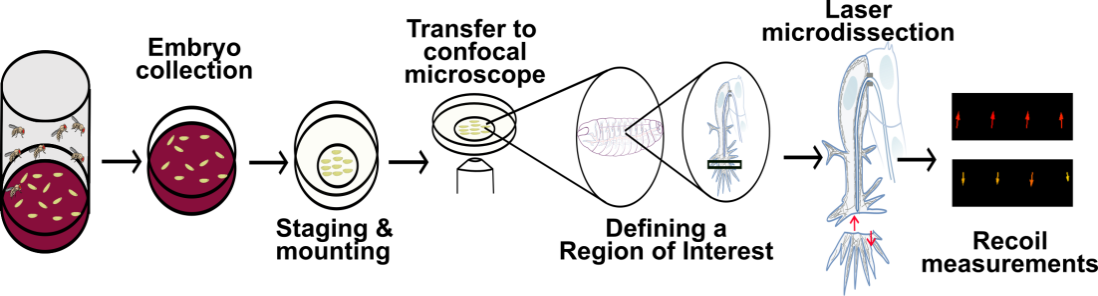

## Background

Tissue morphogenesis requires that cells change their individual shapes and rearrange with one another to form novel structures. Most often, these processes depend on tension and force propagation to induce cell shape changes (Barrera-Velázquez and Ríos-Barrera, 2021; Inman and Smutny, 2021). Forces can act within the same cell or specific subcellular compartments, but can also act in neighboring cells with different biochemical and mechanical properties (Bhide et al., 2021; Ríos-Barrera and Leptin, 2022).

Cytoskeletal elements like actin are often the molecular basis of force generation. Actin fibers interact with motors and crosslinkers to generate force that induces cell autonomous and non-autonomous shape changes (Belmonte et al., 2017; Miao and Blankenship, 2020). Nevertheless, the sole presence of actin meshworks within a cell or tissue may not be indicative of tissue tension. To show that a given structure is under tension, one must manipulate or measure the mechanical properties of the tissue of interest. Different tools have been developed to do this, with particular advantages and caveats. Optogenetics (Izquierdo et al., 2018; Krueger et al., 2019) has a high temporal and spatial specificity but requires the incorporation of genetic tools that are sensitive to light, requiring special handling conditions and restricting imaging possibilities. Atomic force microscopy (Haase and Pelling, 2015) provides a direct readout of tissue properties but uses dedicated equipment and only works in direct contact with the specimen of interest. Fluorescent tension sensors (Lemke et al., 2019) do not require external manipulations but depend on complex image analyses to obtain quantitative information. Laser microdissection was among the first tools that enabled probing mechanical properties of tissues. It consists in focusing high-frequency photons within a region of interest (ROI), which will then disrupt and destabilize actin fibers (Nishimura et al., 2006). Severing the actin cytoskeleton using laser microdissection alters the force distribution in the cell or tissue of interest, resulting in a recoil in the direction in which the tissue is being stretched. Initial recoil speed of the tissue is directly proportional to the tension of the sample immediately before the laser procedure (Rauzi and Lenne, 2014; Shivakumar and Lenne, 2016). Therefore, by measuring the recoil speed, it is possible to infer the tension exerted in the sample of interest. This provides a quantitative approximation of the forces participating in the process of interest and can be used to compare tension generation in different directions (anisotropy), in different regions and stages of embryo development, or in varying genetic conditions (genetic gain- and loss-of-function studies).

There are several ways in which one can measure recoil speed [for an alternative approach, see *Bio-protocol* by Liang et al. (2016)]. In this protocol, we use particle image velocimetry (PIV) analyses, a straightforward approach that does not rely on image segmentation or manual annotation of the signal of interest (Tseng et al., 2012). Instead, PIV compares the spatial distribution of the fluorescent signal within two consecutive time points by comparing the signal within small interrogation windows within the ROI. A shift in the signal in an interrogation window is converted into a vector, which depicts the strength and direction of recoil, if there is any.

As an example of this procedure, we use tracheal terminal cells of the *Drosophila* respiratory system, which uses actin to coordinate their direction of migration with the formation of a subcellular tube (Ríos-Barrera and Leptin, 2022). Nevertheless, this approach can be used to study a wide range of developmental processes in the fruit fly and in other model systems.

## Materials and reagents

1. Strains of *Drosophila melanogaster* expressing the actin-binding domain of Utrophin fused to GFP under the control of a UAS promoter (Flybase ID FBtp0073094) or the ubiquitously-expressed *sqh* promoter [Flybase ID FBtp0073095 (Rauzi et al., 2010; Bhide et al., 2021; Ríos-Barrera and Leptin, 2022)].
2. Paintbrush #3, round (commercially available)
3. Scalpel blade, #23 (e.g., EMS, catalog number: 72049-23)
4. Wash bottle containing distilled water (e.g., VWR, catalog number: 215-4306)
5. Forceps Dumont no. 5 (Merck, catalog number: F6521-1EA)
6. Commercial bleach 50% in water (commercially available)
7. 35 mm glass-bottom dish plates (MatTek, catalog number: P35G-1.5-7-C) or plastic dish plates compatible with confocal microscopy (SPL Life sciences, catalog number: 210350)
8. Halocarbon oil 27 (Sigma-Aldrich, catalog number: H8773-100ML)
9. Heptane glue (see Recipes)
  - 200 mL heptane (Sigma-Aldrich, catalog number: H2198-1L)
  - 3M cellophane, double-sided tape (commercially available)
10. Embryo collection plates (see Recipes, Figure 1A)
  - 6 cm Petri dishes (Sigma-Aldrich, catalog number: P5481-500EA)
  - 250 mL distilled water
  - 10 g agar (Millipore, catalog number: 9002-18-0)
  - 4.2 g sugar molasses or brown sugar (commercially available)
  - 250 mL apple juice (commercially available)
  - 5 mL 10% Nipagin (Merck, catalog number: H5501-100G) in EtOH (Sigma-Aldrich, catalog number: 64-17-5)
  - Dried yeast (commercially available)
  - 100 mL plastic beakers (e.g., Sigma-Aldrich, catalog number: Z245380)
11. Embryo basket (see Recipes, Figure 1B and 1C)
  - 50 mL conical tube (e.g., Corning, catalog number: CLS430828)
  - Stainless steel mesh, 100 wires per inch (e.g., Amazon, Brand LTKJ)
  **Figure 1.**
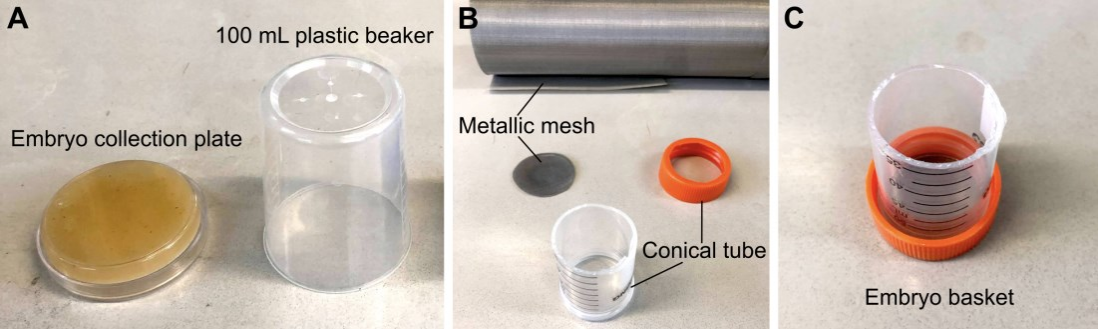
Materials required for embryo collection. (A) 6 cm embryo collection plates and 100 mL plastic beakers are combined to make embryo collection cages. (B) Materials required to make an embryo basket. (C) Assembled embryo basket.

## Recipes

1. **Heptane glue**
  - Mix the tape and heptane for 3 h in a rocker.
  - Discard the tape and use the liquid for downstream applications.
  - Store the heptane glue in a dropper bottle.
2. **Embryo collection plates**
  - Dissolve the agar and brown sugar in 250 mL of water and autoclave.
  - Add 250 mL of apple juice, mix, and add the Nipagin solution.
  - Pour into 6 cm Petri dishes and store at 4 °C.
  - Before using, add yeast paste (dried yeast dissolved in water).
  - Punch small holes on the base of the plastic beakers. These will contain the flies, and the Petri dish with yeast will serve as a lid of the embryo collection cage.
3. **Embryo basket**
  - Make an open-ended 5 cm cylinder by cutting the conical tube using a cutter knife.
  - Cut a round window on the lid of the conical tube.
  - Cut the metallic mesh into a circle that fits inside the lid.
  - Screw in the lid containing the metallic mesh back into the conical tube.

## Equipment

1. Dissecting microscope (e.g., Velab, VE-S7)
2. Laser scanning confocal inverted microscope (e.g., Zeiss, LSM780) with a 63× oil immersion objective, 1.4 NA, and a femtosecond-pulsed infrared laser (Chameleon Compact OPO Fammily, Coherent).

## Software

1. Fiji version 1.53t, https://fiji.sc (Schindelin et al., 2012).
2. PIV analysis plugin version #2020/04/22, https://sites.google.com/site/qingzongtseng/piv (Tseng et al., 2012).

## Procedure

- **Embryo collection**
  1. Set up embryo collection cages by transferring flies of the desired genotype to a plastic beaker. Cover the beaker with a collection plate (see Recipes). This should be done at least three days before the day of the experiment and the plate should be replaced every day. This allows flies to adapt to the new environment and ensures that enough eggs are deposited on the day of the experiment (for additional comments on embryo collection, see General notes and troubleshooting).
  2. One day before the experiment, collect embryos in the embryo cages for 16–24 h (i.e., overnight, Figure 2A–2C).
  3. Replace the embryo plate with a new one.
  4. Take the plate that contains the embryos and add enough bleach to cover the entire surface of the agar. Incubate for 1–2 min or until the chorionic membrane is completely dissolved. You can verify when this is done by looking through the dissection scope. Waggling the embryo plate can also speed up the dissolution of the chorionic membrane.
    Caution: Bleach is corrosive; wear gloves and a lab coat when using it.
  5. Transfer the dechorionated embryos to the embryo basket. To ensure that most embryos are recovered from the collection plate, wash this several times with distilled water using a wash bottle. In addition, wash the embryos with abundant water using tap water (Figure 2D).
    Critical: Embryo exposition to bleach must be as brief as possible. Therefore, it is important to wash the basket with abundant water as soon as the chorionic membrane is dissolved and to do extensive washes to ensure that bleach is completely washed off the embryos. This can be confirmed by smelling the embryo basket.
  6. Using a paintbrush, transfer the dechorionated, washed embryos to a new embryo collection plate (prewarmed to room temperature).
  7. Under the dissecting microscope, collect embryos of the desired stage of development. In this case, take embryos that are at approximately stage 15 of development. In this stage, when looking at the embryos from the dorsal side up, the yolk is no longer heart shaped, but instead has more irregular anterior/posterior edges (Figure 2E–2E’).
    Critical: When selecting the embryos, the light source might increase the temperature of the embryo plate, especially when screening using the bottom light of the microscope. To avoid this, this step should be done as quickly as possible.
    **Figure 2.**
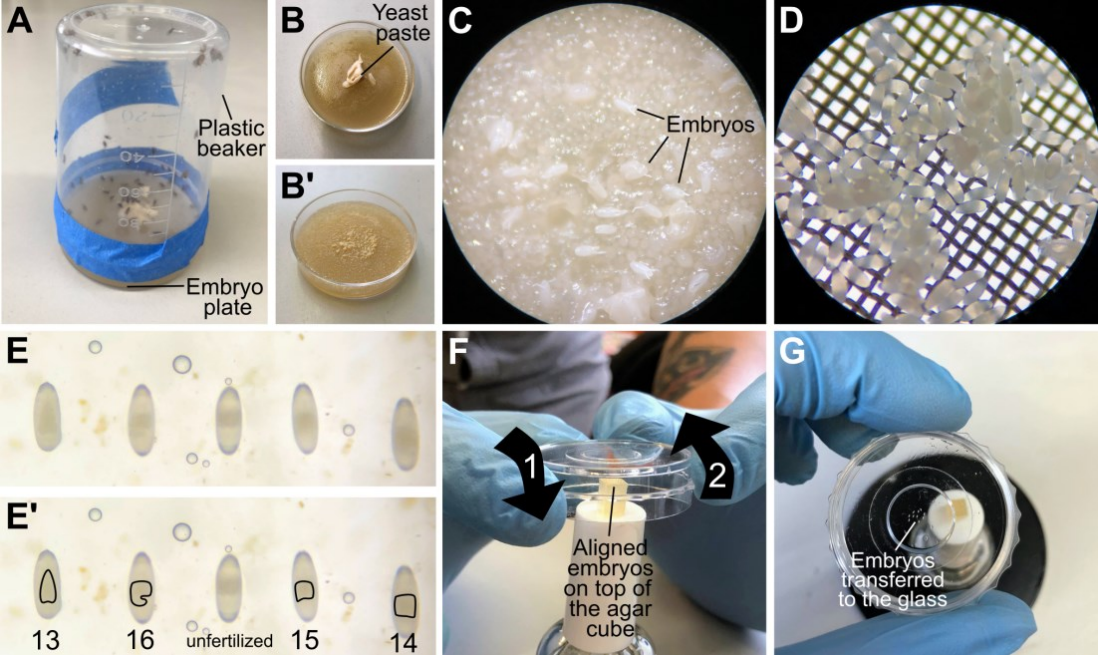
Staging and mounting embryos. (A) Assembled embryo collection cage. (B–B’) Collection plates before (B) and after (B’) embryo collection. (C) Zoom in to an embryo collection plate; embryos are mixed with yeast paste and still have chorionic membrane. (D) Zoom in to an embryo basket, after collecting dechorionated embryos. (E–E’) Embryos aligned in an embryo collection plate, with anterior up and dorsal facing the camera. The shape of the yolk allows staging of the embryos. From left to right: first embryo is in stage 13; the yolk looks like an inverted heart. Second embryo is a stage 16 embryo; the yolk has a two lobed appearance due to folding of the midgut. Third position is an unfertilized egg. The yolk is irregular and off centered, and the rest of the egg is more transparent relative to the other embryos. The fourth embryo is stage 15; the midgut has begun to fold. The last embryo is stage 14; the yolk seems more condensed compared to stage 13 but is more distended than stage 15. (F) All-at-once mounting method. The imaging plate is carefully brought towards the aligned embryos so that they stick to the glass (1). In the same movement, the plate is then moved upwards (2) with the embryos glued to the plate. (G) Embryos attached to the glass after the imaging plate was approached to the agar block.
- **Mounting, one-by-one method**
  1. Pour two drops of heptane glue (See Recipes) into an imaging plate and let the heptane evaporate.
  2. Using the paintbrush, transfer ~5 embryos of the desired stage to the imaging plate, making sure that the region of interest is facing downwards.
    Critical: Embryos should be mounted as quickly as possible to prevent the embryos from dehydrating.
  3. Add enough halocarbon oil to completely cover the embryos. Do not add the oil directly on top of the embryos, as this might detach them from the heptane glue.
- **Mounting, all-at-once method**
  1. Pour two drops of heptane glue into an imaging plate and let the heptane evaporate.
  2. Using forceps, select 5–10 embryos of the desired stage and align them, making sure that the region of interest is facing upwards. Using a blade, cut a cube of agar containing the embryos. Realign them if necessary (Figure 2E).
  3. Invert the imaging plate with the dried heptane glue and put it on top of the embryos so that they attach to the glass (Figure 2F–2G). Because of the side walls of the imaging plate, it is necessary to place the cube of agar containing the embryos on a high surface as exemplified on Figure 2F. In this example, the agar cube with the embryos was placed on top of a nail polish bottle.
    Critical: Avoid putting pressure on the embryos, as this might damage them or make them burst.
  4. Add enough halocarbon oil to completely cover the embryos. Do not add the oil directly on top of the embryos as this might detach them from the heptane glue.
- **Imaging and laser ablation**
  1. In the confocal microscope, focus on one of the embryos of interest. For a detailed protocol on how to perform live imaging of *Drosophila* embryos, refer to Araújo and Llimargas (2023).
  2. Using the bleaching module of the microscope’s software, define a ROI that will be the area intended to cut (Figure 3A–3A’). Set the excitation wavelength at 950 nm using the two-photon laser. Shorter wavelengths (i.e., 850 and 900 nm) give similar results, but 950 nm from a two-photon laser allows to also visualize the GFP actin reporter during the course of the experiment.
    **Figure 3.**
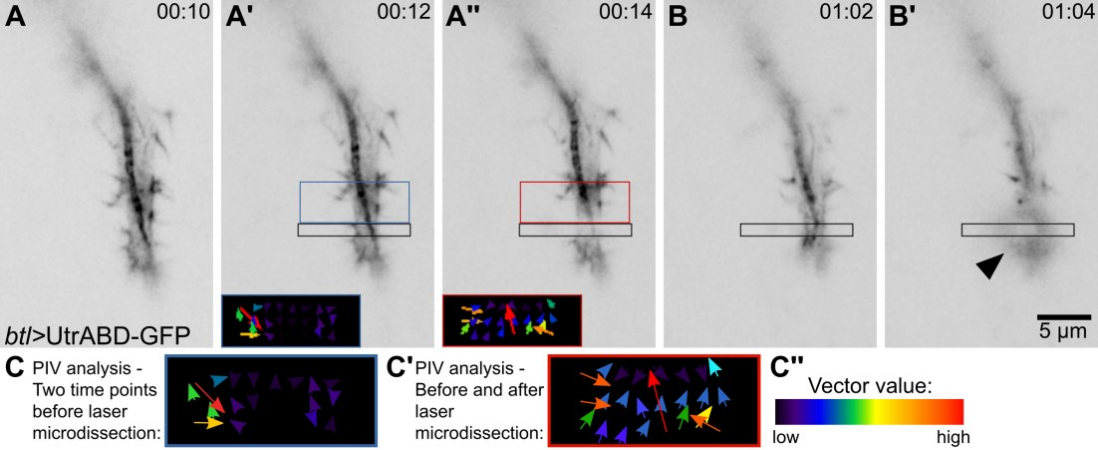
Laser microdissection and recoil measurements. (A–B’) Tracheal terminal cell expressing the actin-binding domain of Utrophin fused to GFP (UtrABD-GFP) under *btl-gal4*. The black box is the ROI where the laser microdissection was or will be done. (A, A’, B) before laser cut; (A’’, B’) after laser cut. (A’) The blue box shows the region where a control particle image velocimetry (PIV) analysis was done, before the laser procedure (time points A vs. A’). (A’’) The red box shows the region where the PIV analysis was done comparing the images before and after laser ablation (time points A’ vs. A’’). Inset shows the graphical representation of the measurements exactly as generated by the plugin. (B’) After a second laser exposition in the same ROI, the sample generates an autofluorescence signal (arrowhead), evidence of phototoxicity. (C–C’’) Higher-resolution reconstructions of the insets shown in (A’–A’’). (C) Control PIV values (from images in A vs. A’). (C’) Experimental PIV values, before and after laser microdissection. (C’’) Color scale for vector values shown in (C–C’).
  3. To calibrate the laser settings, start by using 75% laser power and 8 iterations. If this results in tissue damage, you can lower the number of iterations by half until photo-damage disappears. Tissue damage can be seen as emergence of autofluorescence or permanent tissue deformation (Figure 3B–3B’, Video 1). In contrast, laser power that is not strong enough to induce ablation will only cause bleaching of the fluorescent signal but will not rupture the cytoskeleton.
    **Video 1.**
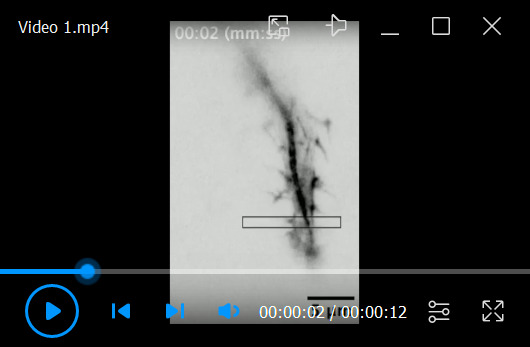
Laser micro-dissection in the tip of a tracheal terminal cell. The black box in (A) and (B) indicates the region where laser micro-dissection was performed. (A) Adequate laser settings. (B) Excessive laser power.
  4. Once the optimal laser settings have been set, move to a different embryo to run the experiment.
  5. Acquire a Z-stack still image of the cell or tissue that you intend to acquire. This will aid as a higher-resolution image of the initial condition of the sample.
  6. Define a new ROI where you intend to induce the laser cut (Figure 3A–3A’). Adjust the bleaching module of the microscope’s software so that the laser cut is done after five frames of imaging and post-acquisition of ~50 frames (total duration of approximately 90 s to acquire the complete recoil, including its plateau phase). Within this time window, acquisition time should be set as fast as possible, with a maximum interval of one image every 10 s.
  7. Immediately after acquisition, you can acquire a new Z-stack still image to record the final morphology of the treated tissue.
  8. Save the image in a lossless file format like .tiff or any other recommended by the microscope software (.lsm, .lif, .nd2).

## Data analysis

**Image analysis**

1. Image processing is done in Fiji. First, make sure that the plugin “iterativePIV” is installed. If it is not, install it by clicking on *Command Menu* > *Help* > *Update*… Then go to *Manage update sites* and then add “https://sites.imagej.net/iterativePIV/” as a new update site. Close *Manage update sites* and click on *Apply changes*. You will have to restart Fiji afterwards. If this does not work, you can directly download the plugin from https://sites.google.com/site/qingzongtseng/ and paste it in the plugins folder within Fiji.
2. Define a ROI that covers the area where you expect to see an effect. In the example used here, we used 107 × 50 pixel (5 μm × 2.3 μm) boxes on one side of the laser cut. Duplicate the ROI for further analyses (Figure 3).
3. The PIV plugin uses two consecutive time points. To measure the initial recoil speed, duplicate into a new image the time points right before and after the laser cut. As negative control, you can use two consecutive time points before the laser cut (Figure 3A and 3C).
4. To run the PIV plugin, you will need to define an interrogation window and a search window size. As suggested in the plugin documentation, you can start by using ¼ of the image dimension as an initial window size and the doubled value as search window size. In this example, we used a 20 pixel window size and 30 pixel search window size.
5. The PIV plugin will generate a graphical representation of the displacements identified (Figure 3A’–3A’’, insets; and 3C–3C’) and a .csv file with multiple values. Average the absolute magnitude values (fifth column) of the different interrogation windows (rows in the file). This is the value we will use as initial recoil speed.
6. As a validation of these results, you can use complimentary image analysis tools, for instance manual tracking of the edges of the ablated area as described elsewhere (Liang et al., 2016).

## General notes and troubleshooting

1. To increase the number of embryos retrieved from embryo collection plates, ensure that the flies are young (1–2 weeks) and that they come from healthy vials. Once in the embryo cages, ensure that the plates always have yeast paste so that the adults are well fed. This can be achieved by replacing the plate with fresh yeast paste three times per day.
2. This protocol explains how to identify embryos at stage 15 of development, when tracheal terminal cells begin to elongate. However, the protocol can be used for other tissues and other developmental stages. To identify the stages of interest the reader might refer to https://www.sdbonline.org/sites/fly/atlas/00atlas.htm (Hartenstein, 1993).
3. A way to recover more embryos of the desired stage is to synchronize embryo collections. To do this, embryos are collected for 30 min. This first collection is not used for experiments in case females had retained eggs, in which case the staging would not be precise. A second 30-min egg collection is done, and the embryos are aged until the time when they are needed. In the example presented here, embryos would be harvested after 10–12 h of incubation. This approach can help beginners to identify the developmental stages of interest for a given experiment.
